# The Comparative Effectiveness of PDE5 Inhibitors and β3 Agonists Versus α-Blockers in Medical Expulsive Therapy for Distal Ureteric Stones: A Systematic Review and Meta-Analysis of Randomized Controlled Trials

**DOI:** 10.7759/cureus.96637

**Published:** 2025-11-11

**Authors:** Maheshwara Ramanah, Indrajit Banerjee, Sarvesh Nunkoo, Jared Robinson, Mooroogiah Krissheeven, Indraneel Banerjee

**Affiliations:** 1 Department of Medicine, Sir Seewoosagur Ramgoolam Medical College, Belle Rive, MUS; 2 Department of Pharmacology, Sir Seewoosagur Ramgoolam Medical College, Belle Rive, MUS; 3 Department of Medicine, Joe Morolong Memorial Hospital, Vryburg, ZAF; 4 Department of Urology, University of Florida Health, Jacksonville, USA

**Keywords:** adrenergic alpha-antagonists, adrenergic beta-3 receptor agonists, tadalafil, tamsulosin hydrochloride, tamsulosin therapy, ureteral calculi

## Abstract

Urolithiasis is a common urinary tract disease. This systematic review and meta-analysis aim to evaluate the effectiveness of alpha-blockers compared to novel therapies. The objective of this systematic review and meta-analysis was to compare the efficacy and safety of emerging pharmacologic therapies, namely tadalafil and mirabegron, with traditional alpha-blockers, such as tamsulosin and silodosin, as medical expulsive therapies (METs) for distal ureteric stones measuring ≤10 or <5 mm. The Preferred Reporting Items for Systematic Reviews and Meta-Analyses 2020 guidelines were implemented during the conduct of this systematic review. A systematic review and meta-analysis of randomized controlled trials was conducted involving adult patients with distal ureteric stones. A total of six studies have been included, and quality assessment has been performed individually. Subgroup analyses were made using forest plots and funnel plots to study the odds ratios (ORs), confidence intervals (CIs), and heterogeneity of the articles. The studies included patients treated with various agents (tamsulosin, silodosin, tadalafil, and mirabegron) for up to four weeks. The primary outcome was the expulsion rate of the stones. Secondary outcomes included stone expulsion time (SET), analgesic use, hospital visits, and adverse effects.

For the stone expulsion rate (SER), in the mirabegron subgroup, an overall pooled OR of 0.98 (95% CI: 0.26-3.66) with high heterogeneity was obtained (I² = 79%, p = 0.02), indicating substantial variability among the included studies. This could be due to the limited number of studies that require further investigation through a sensitivity analysis. In the tadalafil subgroup, an overall pooled OR of 1.79 (95% CI: 0.62-5.14) was obtained with tadalafil showing a better trend toward outcomes compared to alpha-blockers, but the result was not statistically significant. For the secondary outcomes, the tadalafil subgroup showed a significant reduction in the SET compared to alpha-blockers, with a pooled mean difference (MD) of -2.08 days (95% CI: -3.14 to -1.02), indicating no heterogeneity. However, the mirabegron subgroup obtained a pooled MD of 0.16 days (95% CI: -6.06 to 6.38) with very high heterogeneity (I² = 97%, p < 0.0001), suggesting large variability between the studies and no statistically significant difference in expulsion time between mirabegron and alpha-blockers. For the amount of analgesia used, an overall MD of 4.54 mg (95% CI: -53.19 to 62.27) was obtained, indicating no statistically significant difference in analgesic use between the newer drugs and alpha-blockers (p = 0.88). The frequency of adverse effects was noted more in the alpha-blocker group, with significant ejaculation and orthostatic hypertension noted in silodosin only, and insignificant side effects in both the mirabegron and tadalafil groups.

It is concluded from this study that tadalafil is clinically better than alpha-blockers in the MET of distal ureteric stones of <10 mm. Tadalafil has a higher SER and a lower expulsion time, and requires a reduced amount of analgesia. However, given that pooled results are not statistically significant, the following require further evaluation. On the contrary, alpha-blockers are still better than mirabegron, but again, this is not statistically significant enough to prove their supremacy.

## Introduction and background

Urolithiasis is a common urinary tract disease with a lifetime prevalence of 1%-15%, with a peak incidence at the age of 30 years old. Men are two to three times more affected than women [1]. Twenty percent of the calculus in urolithiasis are ureteric stones, and 70% of these stones are present in the distal third of the ureter [2]. Several factors, such as age, sex, race, and geographical region, influence the likelihood of developing a urinary stone [3]. More than 50% of stones smaller than 10 mm pass spontaneously without any medical intervention. If the stone is smaller than 5 mm, the chances of it passing spontaneously are even higher [4]. According to the American Urological Association (AUA), these small stones have a 68% chance of passing, while larger stones have a 47% chance of spontaneous expulsion [5].

Ureteric stones induce spasms of the ureter, which reduce the chance of stone expulsion. Medical therapy is required if patients present with pain and urinary symptoms [2]. If the stones are not passed, they may lead to complications such as UTI, bleeding, hydronephrosis, and even urosepsis [6]. To determine the best course of treatment, the factors that need to be assessed are stone factors, clinical factors, anatomical factors, and technical factors. Stone factors mainly include stone size, number, and location. Other urinary factors include mucosal edema or inflammation [7].

Treatment modalities range from medical to surgical. Extracorporeal shockwave lithotripsy, uteroscopic lithotripsy, and percutaneous nephrolithotomy remain the primary treatment modalities as per European Association of Urology Guidelines [5]. Medical expulsive therapy (MET) is a noninvasive therapy used for ureteric stone expulsion. Many drugs have been tried as MET treatments, including alpha-adrenergic blockers, calcium channel blockers, prostaglandin synthesis inhibitors, phosphodiesterase type 5 inhibitors (PDE5 inhibitors), and steroids. Nowadays, alpha-blockers are used more frequently [8]. The AUA strongly recommends using alpha-blockers (MET) for distal ureteral stones, specifically for stones up to 10 mm. This recommendation is based on evidence that alpha-blockers improve stone expulsion rates (SERs), reduce pain episodes, and decrease hospitalizations. The AUA also notes that the benefit is most likely for stones larger than 5 mm [9]. The ureteric smooth muscles contain large amounts of alpha-1 receptor subtypes (alpha-1a, alpha-1 b, and alpha-1d). The α-1A-receptors predominate in the proximal urethra, prostate, and bladder outflow; the α-1B-receptors are distributed widely in the vascular smooth muscles; and the α-1D-receptors are found predominantly in the intramural ureter and detrusor muscle. The distribution of these receptors in the distal ureter is α-1D > α-1A > α-1B [10]. Alpha-blocker therapy helps in suppressing basal smooth muscle tone, peristaltic frequency, and amplitude. Drugs commonly used are tamsulosin, silodosin, doxazosin, and terazosin. However, some adverse effects, such as anejaculation, nausea, dizziness, and orthostatic hypotension, can occur [11].

PDE5 inhibitors also showed some benefits when used for the expulsion of ureteric stones. They are primarily used for treating erectile dysfunction and pulmonary hypertension. These drugs act by nitric oxide/cyclic guanosine monophosphate (cGMP) signaling pathways, causing smooth muscle relaxation. Tadalafil is an example of such a drug that possesses a long duration of action [12]. Inhibition of the PDE5 isoenzymes results in the intracellular accumulation of cGMP, which activates cGMP-dependent protein kinase, leading to the subsequent phosphorylation of specific substrate proteins. As a second messenger, cGMP plays a central role in signal transduction, regulating smooth muscle relaxation [13].

Another novel drug for stone expulsion involves the use of mirabegron. The latter is a selective beta-3 agonist widely used to treat an overactive bladder. Since beta-3 adrenoreceptors have been found in ureteral smooth muscles and have been found to induce relaxation with almost negligible side effects, they can also be considered an MET [14]. The beta3-AR subtype is widely distributed in human ureteral smooth muscle, and the latter, along with beta2-AR, mediates the ureteral relaxation induced by adrenergic stimulation [15].

While several meta-analyses have evaluated alpha-blockers and PDE-5 inhibitors, few have assessed the efficacy and safety of beta-3 agonists, such as mirabegron, either alone or in comparison with other agents. Recent randomized controlled trials (RCTs) suggest promising results with mirabegron, but this remains inconclusive. Hence, our study clearly highlights this research gap and offers to update the level of evidence regarding the use of emerging drugs, such as mirabegron, in treating distal ureteric stones.

This systematic review and meta-analysis aims to evaluate the effectiveness of alpha-blockers compared to newer therapies such as mirabegron and tadalafil, in treating distal ureteric stones by comparing the SER, the mean expulsion time, and determining the most common adverse effects encountered.

## Review

Methodology

**Figure 1:**
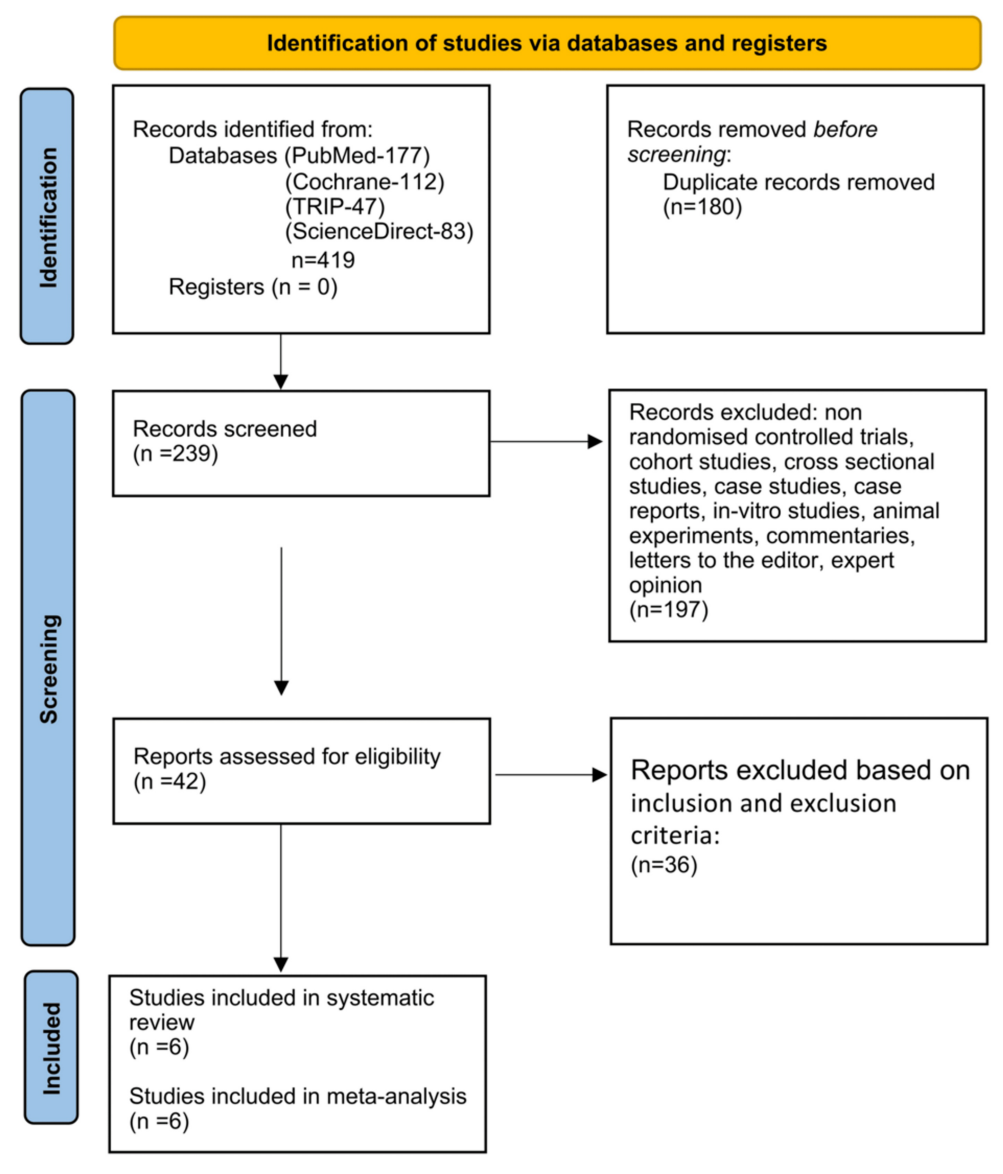
PRISMA 2020 flow diagram PRISMA: Preferred Reporting Items for Systematic Reviews and Meta-Analyses

This systematic review and meta-analysis was performed with a frequentist approach. Preferred Reporting Items for Systematic Reviews and Meta-Analyses 2020 guidelines were implemented during the conduction of this systematic review (Figure 1) [16].

Literature search

An extensive review of the literature was conducted using the following databases: PubMed, Cochrane Central Register of Controlled Trials, ScienceDirect, and the Trip Medical Database to identify relevant manuscripts (Table 1). The search string used for the literature search was based on the Patient, Intervention, Control, and Outcome guidelines and was conducted from June 2016 to September 2025. The literature search language was limited to English only, given that the level of evidence from non-English trials was negligible. This may present as a limitation in the form of language bias. A combination of keywords and Boolean operators was used for data extraction: (("Ureteral Calculi"[Title/Abstract] OR "Ureteric Stones"[Title/Abstract] OR "Ureterolithiasis"[Title/Abstract]) AND ("Tamsulosin"[Title/Abstract] OR "Adrenergic alpha-Antagonists"[Title/Abstract] OR "Alpha blockers"[Title/Abstract] OR "Tadalafil"[Title/Abstract] OR "Phosphodiesterase 5 Inhibitors"[Title/Abstract] OR "Mirabegron"[Title/Abstract] OR "Adrenergic beta-3 Receptor Agonists"[Title/Abstract])). The following filters were applied: the range of years applied was from June 2016 to September 2025, randomized controlled trials only, and English-based articles only. The strategy used in the search, along with the total number of articles screened, is shown in Table 1.

**Table 1:** Search strategy

Databases searched	Boolean operators and keywords	Number of articles
PubMed	(("Ureteral Calculi"[Title/Abstract] OR "Ureteric Stones"[Title/Abstract] OR "Ureterolithiasis"[Title/Abstract]) AND ("Tamsulosin"[Title/Abstract] OR "Adrenergic alpha-Antagonists"[Title/Abstract] OR "Alpha blockers"[Title/Abstract] OR "Tadalafil"[Title/Abstract] OR "Phosphodiesterase 5 Inhibitors"[Title/Abstract] OR "Mirabegron"[Title/Abstract] OR "Adrenergic beta-3 Receptor Agonists"[Title/Abstract]))	177
Cochrane Central Register of Controlled Trials	(("Ureteral Calculi"[Title/Abstract] OR "Ureteric Stones"[Title/Abstract] OR "Ureterolithiasis"[Title/Abstract]) AND ("Tamsulosin"[Title/Abstract] OR "Adrenergic alpha-Antagonists"[Title/Abstract] OR "Alpha blockers"[Title/Abstract] OR "Tadalafil"[Title/Abstract] OR "Phosphodiesterase 5 Inhibitors"[Title/Abstract] OR "Mirabegron"[Title/Abstract] OR "Adrenergic beta-3 Receptor Agonists"[Title/Abstract]))	112
TRIP database	(("Ureteral Calculi"[Title/Abstract] OR "Ureteric Stones"[Title/Abstract] OR "Ureterolithiasis"[Title/Abstract]) AND ("Tamsulosin"[Title/Abstract] OR "Adrenergic alpha-Antagonists"[Title/Abstract] OR "Alpha blockers"[Title/Abstract] OR "Tadalafil"[Title/Abstract] OR "Phosphodiesterase 5 Inhibitors"[Title/Abstract] OR "Mirabegron"[Title/Abstract] OR "Adrenergic beta-3 Receptor Agonists"[Title/Abstract]))	47
ScienceDirect	(("Ureteral Calculi"[Title/Abstract] OR "Ureteric Stones"[Title/Abstract] OR "Ureterolithiasis"[Title/Abstract]) AND ("Tamsulosin"[Title/Abstract] OR "Adrenergic alpha-Antagonists"[Title/Abstract] OR "Alpha blockers"[Title/Abstract] OR "Tadalafil"[Title/Abstract] OR "Phosphodiesterase 5 Inhibitors"[Title/Abstract] OR "Mirabegron"[Title/Abstract] OR "Adrenergic beta-3 Receptor Agonists"[Title/Abstract]))	83
Total		419

Eligibility criteria

Inclusion Criteria

All RCTs providing information on ureteric stones, newer drugs, alpha-blockers, and stone size, as well as full-text articles published between June 2016 and September 2025 and available in English, were assessed and included in the study. Adult patients aged 18 years and above, with a confirmed diagnosis upon imaging (plain X-ray film of the kidney, ureter, and bladder, computed tomography scan, intravenous pyelography, and ultrasonography), a single stone measuring 10 mm or smaller, and located in the distal ureter, were included in this study.

Exclusion Criteria

Data resources, including nonrandomized controlled trials, cohort studies, case-control studies, cross-sectional studies, case series, case reports, in vitro studies, animal experiments, commentaries, letters to the editor, expert opinions, and review articles, were excluded from this systematic review. Patients with bilateral stones, evidence of a UTI, or hydronephrosis, along with complicating factors such as sepsis, uncontrollable pain, or deterioration of renal function, were excluded. Additionally, individuals with kidney or ureteral abnormalities, such as having a single kidney or ureteral malformations, as well as pregnant or lactating women, were excluded from the study.

Types of outcome measures

The primary outcome measure included SER. The secondary outcome measures include stone expulsion time (SET), analgesic requirement (nonsteroidal anti-inflammatory drugs (NSAIDs) dose/duration), and adverse drug effects such as retrograde ejaculation, headache, and hypotension.

Data synthesis

Data extraction was conducted on the relative titles. The titles were initially examined based on their abstracts. Thereafter, the full texts of the examined RCT titles that met the eligibility requirements were considered for the final selection. The literature evaluation was independently performed by MR and SN, and discrepancies were reconciled by MK, JR, and IB. The extracted data included the study authors, year, country, sample size, use of newer drugs, alpha-blockers, stone size in millimeters, location, SERs (%), mean expulsion time (days), analgesic use (mg), and adverse drug reactions.

Risk of bias assessment

The risk of bias was assessed by three independent researchers, MR, SN, and MK, and reconciliation of discrepancies was performed by the senior author IB. The Cochrane Risk-of-Bias tool for randomized trials (RoB2) was used for quality assessment [17]. The RoB2 tool is best suited and implemented to assess the domains at low, unclear, and high risk of bias. The Risk-of-bias Visualization tool is a web-based application used to generate traffic light plots and weighted bar plots for risk of bias summaries [18].

Statistical analysis

Measures of the Treatment Effect

We analyzed outcomes as continuous or dichotomous data using standard statistical techniques with a random-effects model up to the end of follow-up [19]. For dichotomous outcomes, we used the odds ratio (OR) and 95% confidence interval (CI). For continuous outcomes (mean expulsion time and analgesic use), the mean difference (MD) was calculated with the 95% CI. Predefined subgroup analyses were conducted based on comparator type (mirabegron vs. tadalafil), which explored the impact of study sample size, weight of study, and study quality, with a p value set at <0.05, and analyses were performed using Review Manager (RevMan) version 8.11 (The Cochrane Collaboration, London, UK).

Assessment of Heterogeneity

Sensitivity analyses were conducted to evaluate the robustness of the pooled results. These included leave-one-out analyses (excluding one study at a time) as well as exclusion of studies with a high risk of bias. Additionally, results were compared using random-effects models. The overall conclusions remained stable across all sensitivity analyses, confirming the reliability of the findings. The I^2^ statistic and χ^2^ test were used to measure heterogeneity among studies in each analysis, employing a random-effects model. Heterogeneity was categorized as low (<30%), moderate (30%-60%), or high (>60%) based on the I^2^ values [15]. If substantial heterogeneity was identified, it was reported, and possible causes were explored by performing prespecified subgroup analyses (SER (%), mean expulsion time (days), analgesic use (mg), and study design (RCT)). Outcome measures were cross-validated using the MD and OR. Furthermore, meta-regression was applied to assess the relationships between newer drugs, alpha-blockers, weight, and control SER and the primary outcome using RevMan version 8.1.1, Cochrane Collaboration (2024) software [20].

Results

The literature search produced 419 articles. Among these, 180 were noted as duplicates and excluded from the initial analysis. Therefore, 239 manuscripts were screened after deduplication. Abstracts, case studies, reports, editorials, viewpoints, cross-sectional studies, cohort studies, case-control studies, case series, and letters to the editor/correspondence manuscripts (n = 197) were additionally excluded. A total of 42 full-text articles were assessed for eligibility. An in-depth evaluation and analysis further excluded 36 articles from the analysis due to wrong population, inappropriate comparator, insufficient data, and study design issues, and also based on the inclusion and exclusion criteria. Six RCTs were finally assessed regarding the comparative effectiveness of emerging pharmacologic therapies vs. alpha-blockers in the MET of distal ureteric stones. Authors, year, country, sample size, newer drugs, alpha-blockers, stone size in millimeters, location, SERs (%), mean expulsion time (days), analgesic use (milligrams), and adverse drug reactions have been depicted in Tables 2-4.

**Table 2:** Type of study, country, sample size (n), newer drugs, alpha-blockers, stone size, and location of stone RCT: randomized controlled trials Funding sources, duration of therapy, and imaging modality used for confirmation of stone expulsion were not reported in the table

Study	Type of study	Country	Sample size (n)	Newer drugs	Alpha-blockers	Stone size	Location
Samir et al. [21]	RCT	Egypt	180	Mirabegron 50 mg	Silodosin 8 mg	5-10 mm	Distal ureter
Abdel-Kader et al. [22]	RCT	Egypt	105	Mirabegron 50 mg	Silodosin 8 mg	≤10 mm	Distal ureter
Abdelaal et al. [23]	RCT	Egypt	150	Tadalafil 5 mg	Silodosin 8 mg	≤10 mm	Distal ureter
Falahatkar et al. [24]	RCT	Iran	132	Tadalafil 10 mg	Tamsulosin 0.4 mg	5-10 mm	Distal ureter
Tang et al. [25]	RCT	China	90	Mirabegron 50 mg + tamsulosin 0.4 mg	Tamsulosin 0.4 mg	≤5 mm	Distal ureter
Kc et al. [26]	RCT	Nepal	85	Tadalafil 0.4 mg	Tamsulosin 0.4 mg	5-10 mm	Distal ureter

**Table 3:** Stone expulsion rates (%), mean expulsion time (days), and analgesic use (mg) NA: not available

Study	Stone expulsion rates (%)		Mean expulsion time (days)		Analgesic use (mg)	
	Newer drugs	Alpha-blockers	Newer drugs	Alpha-blockers	Newer drugs	Alpha-blockers
Samir et al. [21]	38.6%	61%	12.6 ± 4.5	9.25 ± 3.9	131.7 ± 57.6	126.6 ± 44.2
Abdel-Kader et al. [22]	51.4%	57.1%	11 ± 3.1	14 ± 2.3	NA	NA
Abdelaal et al. [23]	90%	76%	8.7 ± 3.3	11.3 ± 4.2	120 ± 55.3	163 ± 77.5
Falahatkar et al. [24]	63.6%	72.7%	21.13 ± 1.17	17.75 ± 75	270.45 ± 170.9	165.9 ± 219.6
Tang et al. [25]	91%	67%	NA	NA	NA	NA
Kc et al. [26]	84.1%	61%	8.08 ± 3.3	9.64 ± 3.8	120.4 ± 201.8	146.3 ± 245.0

**Table 4:** Adverse drug reactions

Study	Adverse drug reactions
Samir et al. [21]	Retrograde ejaculation was noted in silodosin (83.3%) only
Abdel-Kader et al. [22]	Significant ejaculation and orthostatic hypertension were noted in silodosin only
Abdelaal et al. [23]	Abnormal ejaculation and orthostatic hypertension were noted in silodosin only
Falahatkar et al. [24]	No such adverse effects were observed in both drugs
Tang et al. [25]	Very low side effects were encountered in both drugs
Kc et al. [26]	Postural hypertension was more in tadalafil than in tamsulosin

Risk of bias assessment

**Figure 2:**
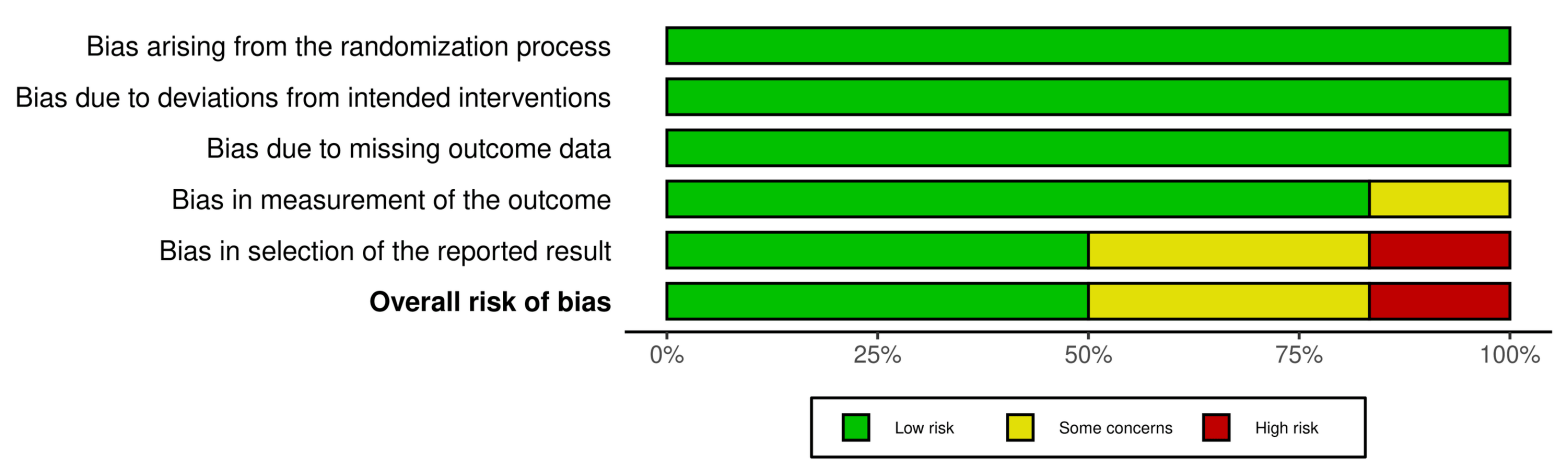
Summary of risk of bias for RCTs (weighted bar plots) RCTs: randomized controlled trials

**Figure 3:**
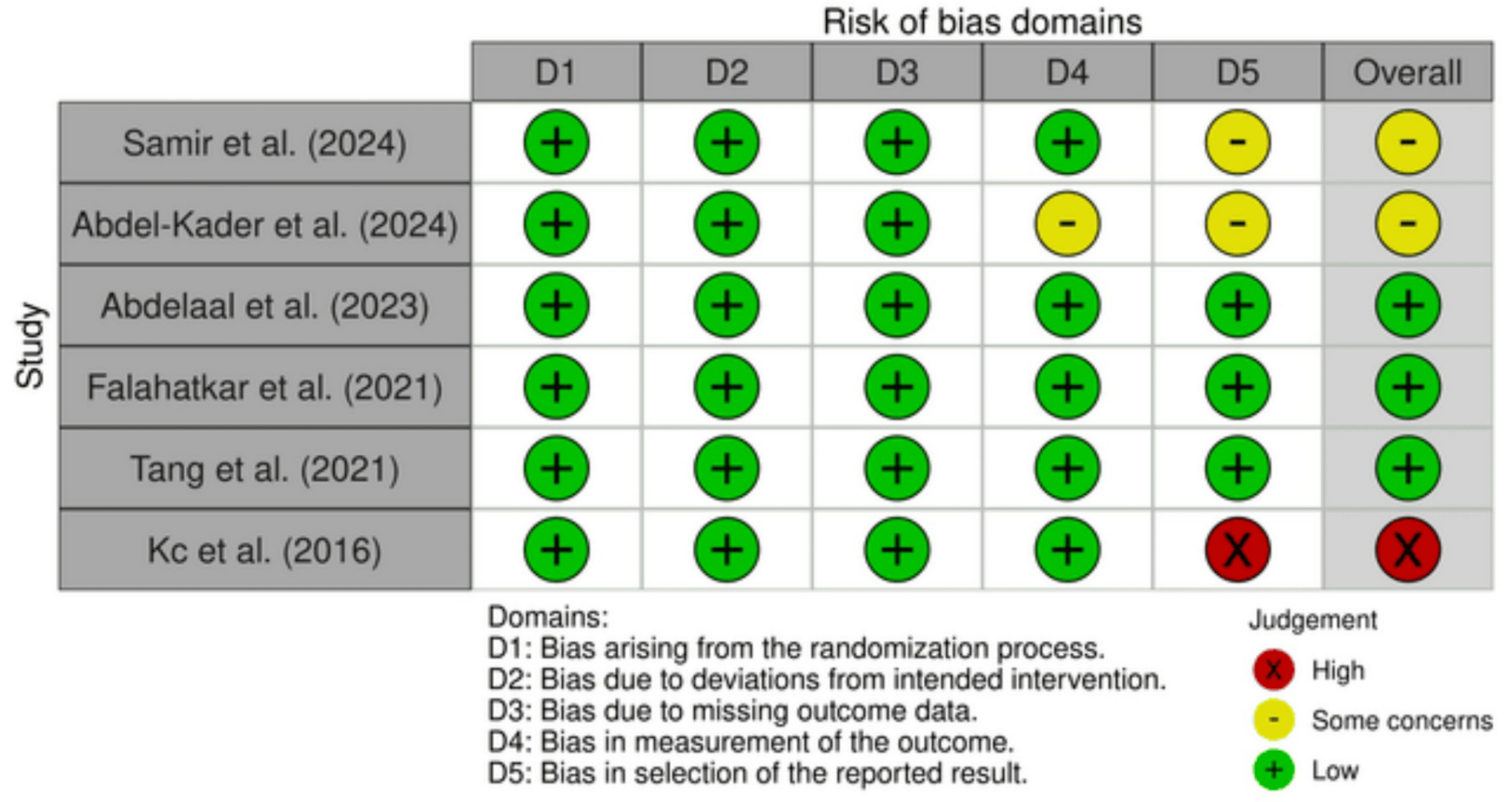
Risk of bias of RCTs (traffic light plot) RCTs: randomized controlled trials Source: [21-26]

All the included RCTs underwent a quality assessment via the ROB2 tool. The results thereof indicate a low risk of bias in the randomization process (low risk 100%), deviations from intended interventions (low risk 100%), missing outcome data (low risk 100%), measurement of outcome (low risk 83.3%, some concern 16.7%), and selection of reported result (low risk 49.9%, some concerns 33.4%, and high risk 16.7%). The overall risk of bias for the six RCTs was found to be as follows: low risk, 49.9%; some concerns, 33.4%; and high risk, 16.7%, indicating some concerns (Figures 2, 3).

Primary outcome

**Figure 4:**
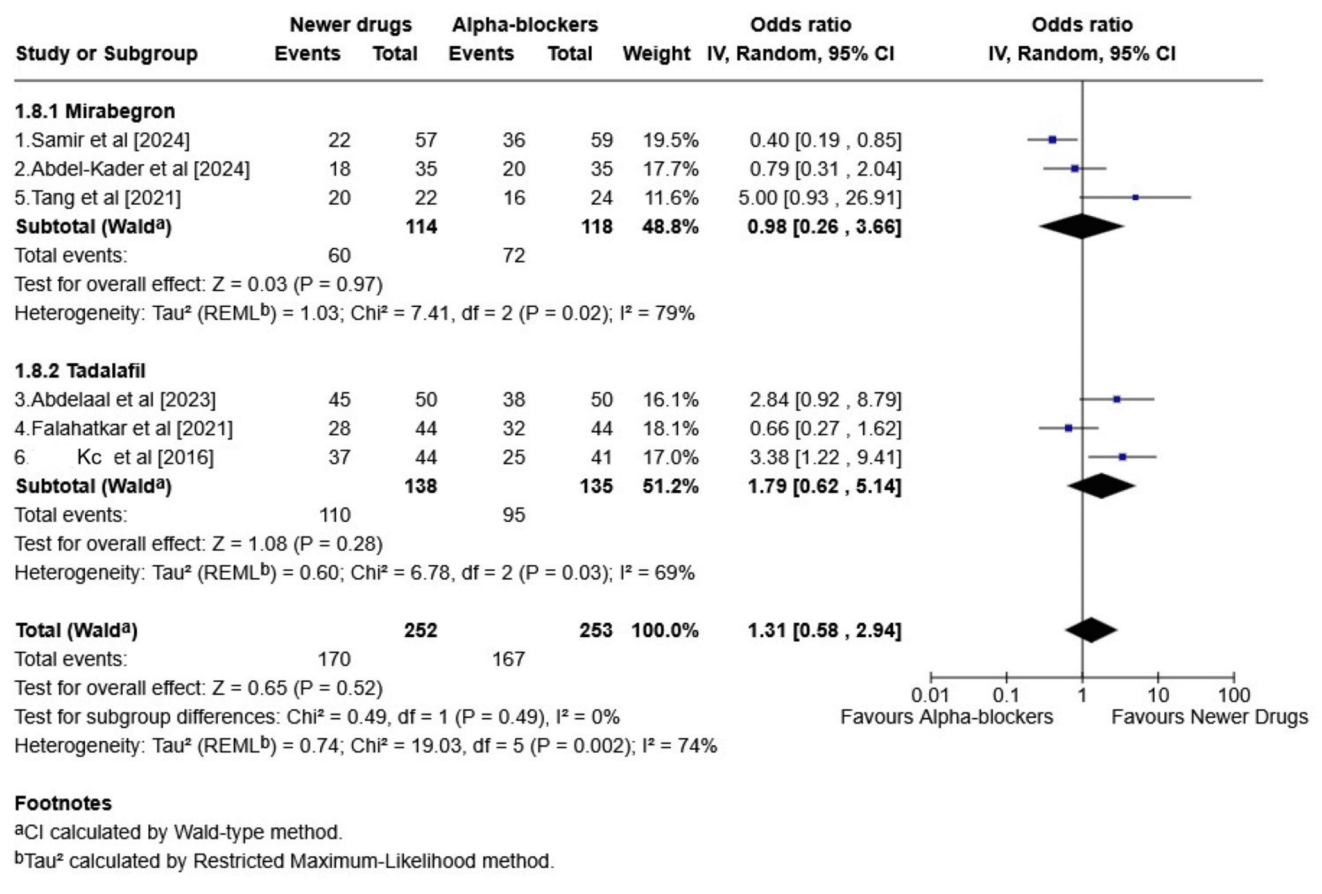
Newer pharmacological drugs (mirabegron and tadalafil) vs. traditional alpha-blockers in the stone expulsion rate of ureteric stones (forest plot) CI: confidence interval; REML: restricted maximum likelihood Source: [21-26]

This forest plot presents a meta-analysis comparing the effectiveness of newer pharmacological drugs (mirabegron and tadalafil) vs. traditional alpha-blockers in the SER of ureteric stones (Figure 4).

Mirabegron Subgroup

The pooled OR was found to be 0.98 (95% CI: 0.26-3.66), indicating no significant difference in SERs between mirabegron and alpha-blockers. High heterogeneity (I² = 79%, p = 0.02) was mainly due to differences in sample size. No sensitivity analysis was performed to address this.

Tadalafil Subgroup

The overall pooled OR was found to be 1.79 (95% CI: 0.62-5.14), indicating tadalafil may have a trend toward better outcomes compared to alpha-blockers. Although the result was not statistically significant, clinically, tadalafil shows equal SER to alpha-blockers (tamsulosin). Heterogeneity was found to be moderate to high (I² = 69%, p = 0.03), indicating considerable variability among the included studies.

Overall Effect (All Newer Drugs vs. Alpha-Blockers)

The combined OR was found to be 1.31 (95% CI: 0.58-2.94). Overall heterogeneity was substantial (I² = 74%, p = 0.002), suggesting notable variability among the studies.

Secondary outcomes

**Figure 5:**
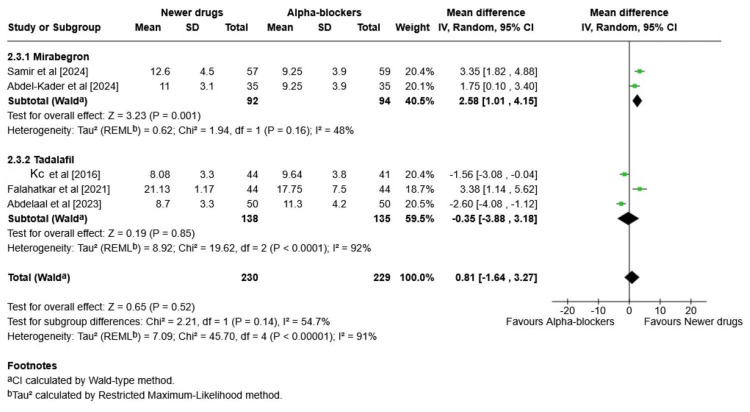
Mean expulsion time (in days) for ureteric stones between novel pharmacologic agents (mirabegron and tadalafil) and alpha-blockers (forest plot) SD: standard deviation; CI: confidence interval; REML: restricted maximum likelihood Source: [21-24,26]

This meta-analysis compares the mean expulsion time (in days) for ureteric stones between novel pharmacologic agents (mirabegron and tadalafil) and alpha-blockers (Figure 5).

Mirabegron Subgroup

The pooled MD was found to be 0.16 days (95% CI: -6.06 to 6.38), indicating no statistically significant difference in expulsion time between mirabegron and alpha-blockers. Heterogeneity was found to be very high (I² = 97%, p < 0.0001), suggesting large variability between the studies.

Tadalafil Subgroup

The pooled MD was found to be -2.08 days (95% CI: -3.14 to -1.02). Tadalafil significantly reduces SET compared to alpha-blockers. Heterogeneity was found to be low (I² = 0%), indicating consistency in the included studies.

Overall Effect (All Newer Drugs vs. Alpha-Blockers)

Overall, the total pooled MD was found to be -0.90 days (95% CI: -3.71 to 1.91). The overall reduction in expulsion time with newer drugs is not statistically significant. Heterogeneity was found to be very high (I² = 92%, p < 0.00001), driven largely by the inconsistency within the mirabegron subgroup.

Tadalafil shows a statistically significant reduction in SET compared to alpha-blockers. Mirabegron shows no significant advantage and has a high variability in results. Therefore, while newer drugs show a trend toward faster stone passage, the pooled difference is not statistically significant due to heterogeneity, particularly from the mirabegron subgroup. Tadalafil remains the more promising newer agent for reducing the SET.

Pooled Results for the Analgesic Use

**Figure 6:**
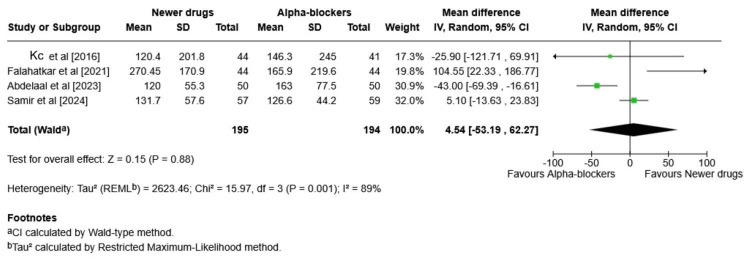
Analgesic use (mg) forest plot SD: standard deviation; CI: confidence interval; REML: restricted maximum likelihood Source: [21,23,24,26]

Overall, MD was found to be 4.54 mg (95% CI: -53.19 to 62.27). There was no statistically significant difference in analgesic use between the newer drugs and alpha-blockers (p = 0.88). Heterogeneity was found to be I² = 89% (p = 0.001), mostly because of the different doses of analgesics used in each group. This high heterogeneity among the studies indicates that the pooled results' reliability is low. The overall analysis shows no significant difference in analgesic use between newer pharmacologic agents and alpha-blockers in patients undergoing MET for ureteric stones. However, some subgroup conclusions are not statistically significant (Figure 6).

Publication bias and funnel plots

**Figure 7:**
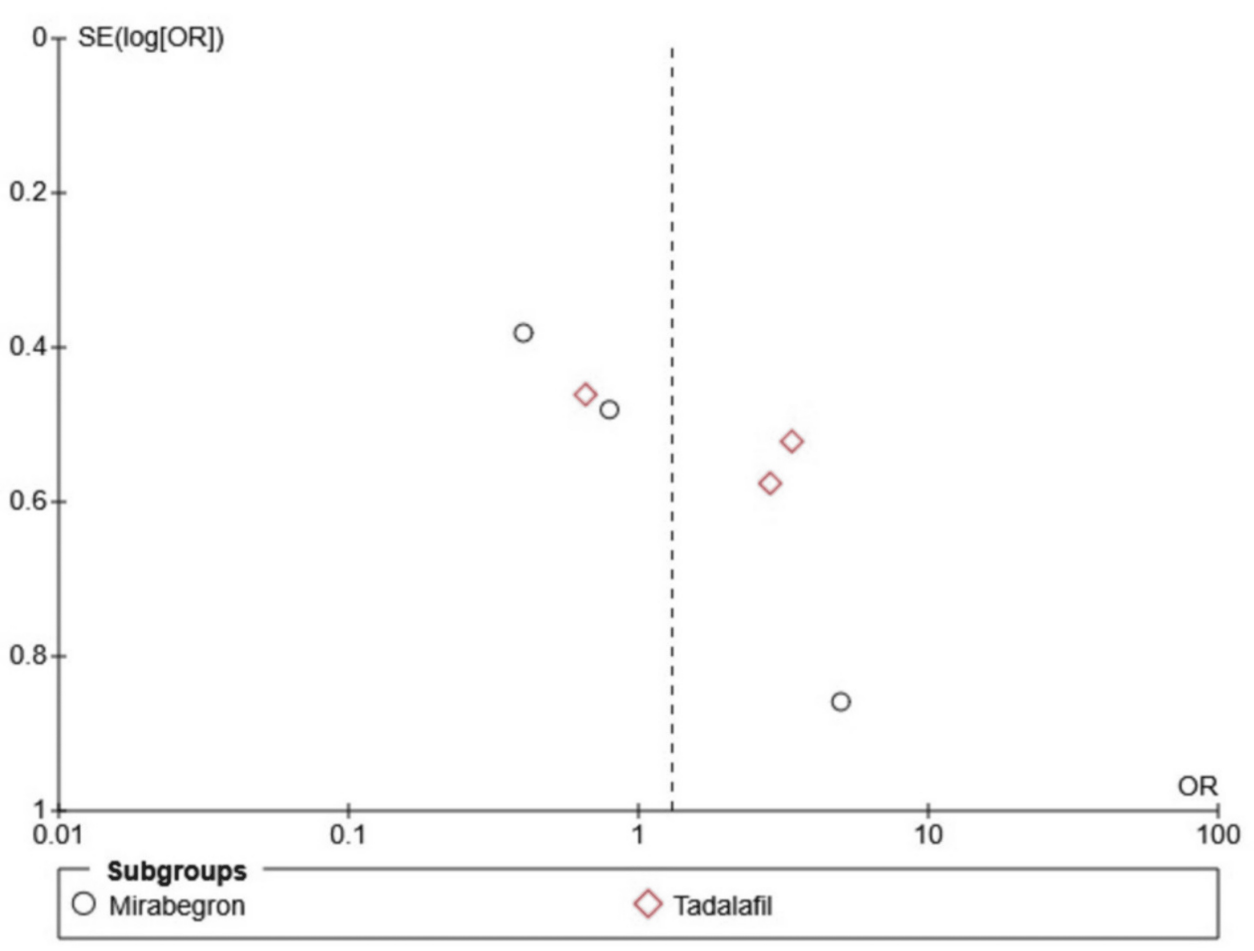
Funnel plot showing stone expulsion rate SE: standard error; OR: odds ratio

**Figure 8:**
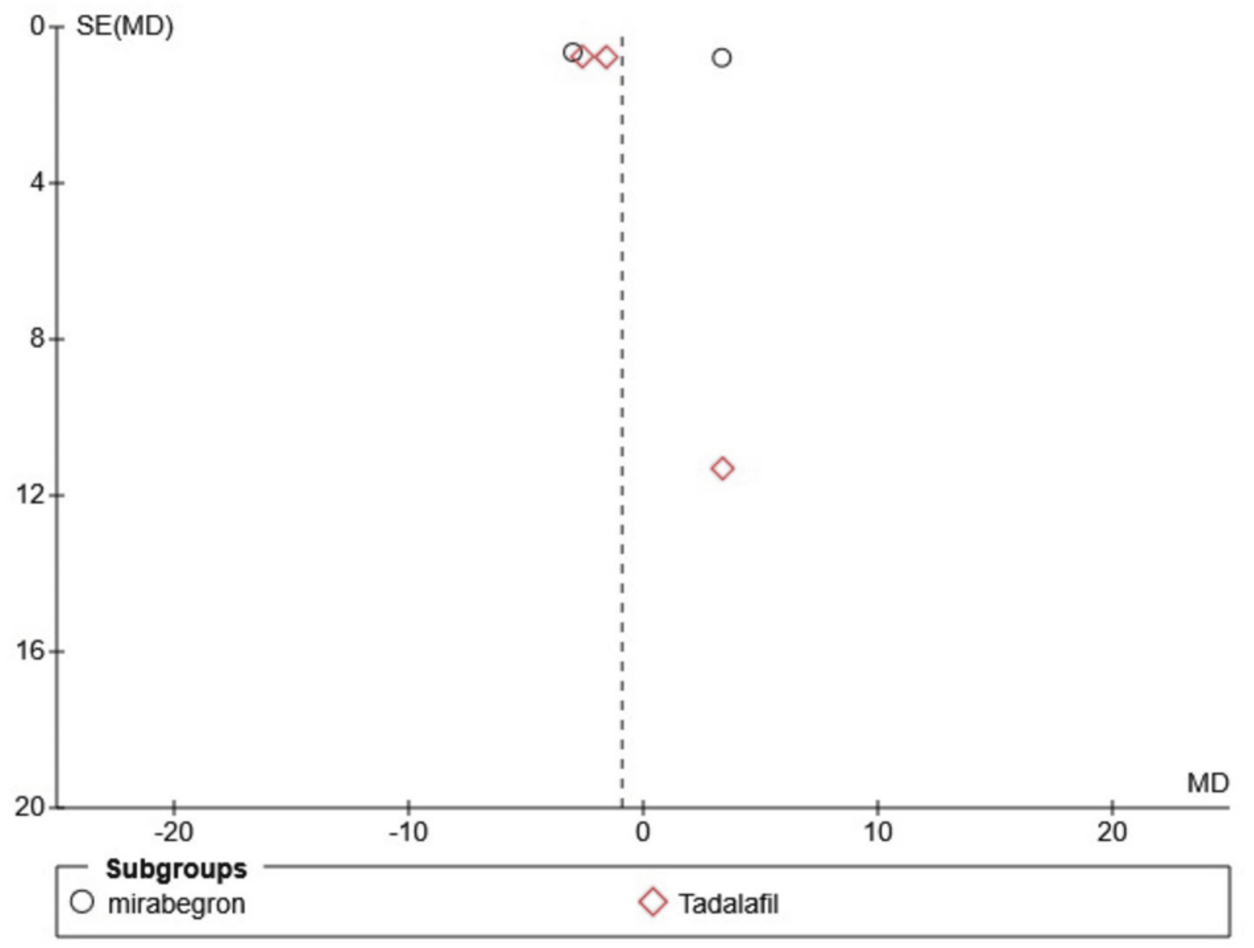
Funnel plot showing the mean expulsion interval SE: standard error; MD: mean difference

**Figure 9:**
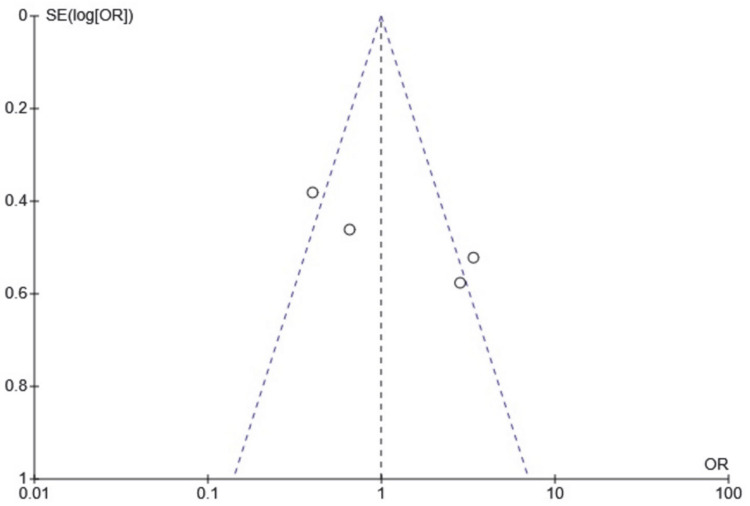
Funnel plot showing analgesia use SE: standard error; OR: odds ratio

The findings remain consistent in the sensitivity analysis, though the funnel plot indicated possible publication bias. As a limitation, the funnel plots were presented without a formal test, and given that only six studies were included in the funnel plot, it is challenging to accurately assess asymmetry, which may lead to misinterpretation (Figures 7-9).

Discussion

Mirabegron, a beta-3 adrenergic agonist, relaxes the smooth ureteral muscles, thus helping the passage of kidney stones [14]. Mirabegron is also used to treat overactive bladder and neurogenic detrusor overactivity [27]. Likewise, tadalafil, a phosphodiesterase-5 inhibitor, causes smooth muscle relaxation through nitric oxide/cGMP signaling pathways [12]. Tadalafil is also used to treat pulmonary hypertension and erectile dysfunction [28]. These drugs are used as novel treatment modalities against the conventional alpha-blockers in the MET of distal ureteric stones. Hence, our systematic review and meta-analysis consists of assessing the SER, mean expulsion time, and amount of analgesia used, as well as evaluating whether the results obtained are statistically significant.

Samir et al. conducted an RCT on 180 subjects, where mirabegron was evaluated as a newer treatment modality against silodosin and a control drug in the MET of distal ureteric stones. Sixty patients were assigned to each group and assessed for SER as a primary outcome for a maximum of four weeks. The study demonstrated that silodosin had a significantly higher SER of 61% compared to mirabegron, at 38.6%. As secondary outcomes, the SET was also found to be shorter in silodosin (9.25 ± 3.9 days) than in mirabegron (12.6 ± 4.5 days), and the amount of analgesia used was 126.6 ± 44.2 mg in silodosin compared to 131.7 ± 57.6 mg in mirabegron [21]. A similar study conducted by Bayar et al. showed that the SER was better with silodosin at 64.8% compared to mirabegron at 52.5%. Moreover, silodosin had a smaller mean expulsion interval (7.7 ± 4.5 days) and required more analgesic use (2.73 ± 2.1 mg) compared to mirabegron, with 9.8 ± 4.7 days and 2.23 ± 1.8 mg, respectively [29].

Furthermore, the RCT conducted by Abdel-Kader et al. included 105 patients, and it was found that silodosin had a higher expulsion rate (57.1% vs. 51.4%) and a higher mean expulsion interval (14 ± 2.3 vs. 11 ± 3.1 days) [22]. Similarly, Sharma et al. found a more successful expulsion rate with silodosin (84.3%) compared to mirabegron (77.5%). Patients in the silodosin group had a significantly shorter time for stone expulsion (mean 10.0 ± 5.3) compared to those in the mirabegron group (mean 15.7 ± 7.1) (p = 0.0004) [30]. On the other hand, a study conducted by Tang et al. enrolled 90 participants, with 45 receiving mirabegron 50 mg plus tamsulosin 0.2 mg, and the other 45 receiving tamsulosin 0.2 mg alone. The frequency of expelled stones was 90.9% in the study group and 66.7% in the control group, with a p value of 0.046 after treatment [25]. Mirabegron, combined with tamsulosin, produces synergistic actions by relaxing the detrusor smooth muscle through β3-adrenoceptor and by directly reducing the ureteral smooth muscle tone through α1A-selective adrenergic blocker, respectively. Silodosin is clearly better than mirabegron, but when it comes to the other alpha-blocker, that is tamsulosin, mirabegron should be considered a more viable option for stone expulsion.

However, in a study conducted by Abdelaal et al., where 150 participants were assessed, tadalafil showed a statistically increased SER compared to silodosin (90% vs. 76%; p = 0.043), a lower mean expulsion time of 8.7 ± 3.3 vs. 11.3 ± 4.2 days and significantly less analgesia use of 120 ± 55.3 vs. 163 ± 77.5 mg with silodosin [23]. The following was again proved by Rahman et al., where 120 participants were randomized among three groups, and among these, a combination of tadalafil 5 mg plus silodosin 8 mg was given to 40 patients, and another 40 people received silodosin 8 mg alone. Results showed that the combination therapy had a higher expulsion rate of 90% vs. 77.5% (p = 0.05) and a lower mean expulsion interval of 12 ± 2.2 vs. 15±3.3 days compared to silodosin monotherapy [31]. Hence, as suggested by both studies, tadalafil is a better option to consider for distal ureteric stones compared to silodosin, and this emerging drug can replace conventional alpha-blockers.

Now, we compare tadalafil with another commonly used alpha-blocker, namely tamsulosin. Data produced by Kc et al. showed the following findings. The study consisted of 85 participants, where group A had 41 patients receiving tamsulosin 0.4 mg and group B had 44 patients receiving tadalafil 10 mg at bedtime for two weeks. Data showed that the frequency of stone expulsion was significantly higher in the tadalafil group than in the tamsulosin group (84.1% vs. 61.0%, p = 0.017). The mean expulsion interval was also lower in the tadalafil group (8.08 ± 3.3 vs. 9.64 ± 3.8 days), but this difference was not significant (p = 0.094), and the amount of analgesia used was greater in group A patients (146.3 ± 245.0 vs. 120.4 ± 201.8 mg) [26]. A similar study was conducted by Laddha et al., where 150 patients were enrolled and divided equally into three groups. Group A received a placebo, group B received tadalafil 10 mg OD, and group C received tamsulosin 0.4 mg OD. It was found that 80% (40 of 50 patients) in the tadalafil group and 74% (37 of 50 patients) in the tamsulosin group experienced successful stone expulsion; however, this difference was not statistically significant (p = 0.139). The mean expulsion time was 7.21 ± 3.29 days for the tadalafil group and 8.32 ± 3.14 days for the tamsulosin group. Pertaining to the amount of analgesia used (diclofenac), there was a significantly lower use in the tadalafil group (132.93 ± 82.62) compared to the tamsulosin group (277.08 ± 103.12) [32].

Falahatkar et al. conducted an RCT in 132 patients where group A consisted of 44 patients on tamsulosin 0.4 mg, group B had 44 patients receiving tadalafil 10mg once daily, and group C was given a placebo for up to four weeks. The SER for tamsulosin was 72.7%, and for tadalafil, it was 63.6%; however, the results were not significant (p = 0.294). Again, the mean expulsion time was lower in tamsulosin than in tadalafil (17.75 ± 7.5 vs. 13 ± 1.17 days), but these differences did not reach statistical significance (p = 0.46). Additionally, the mean dose of used NSAIDs was much less with tamsulosin (818.18 ± 618.05 vs. 1,068.02 ± 503.3 mg) [24].

In this meta-analysis, for the primary outcome, the SER was evaluated for both mirabegron and tadalafil as newer treatment modalities and was compared to alpha-blockers, namely tamsulosin and silodosin. For the mirabegron subgroup, a pooled OR of 0.98 (0.26-3.66) was obtained, which signifies a slight advantage of alpha-blockers over the new drug, but the values are not statistically significant (p = 0.97). Additionally, I² = 79% indicates high heterogeneity, meaning that the results vary substantially across studies. With tadalafil, a pooled OR of 1.79 (0.62-5.14) (p = 0.28) and I² = 69% were obtained, which suggests potential benefit of tadalafil over alpha-blockers, but again not significant with moderate to high heterogeneity. Similarly, a meta-analysis conducted by Yan et al. compared the safety and effectiveness of alpha-blockers with those of mirabegron. The SER was found to be significantly greater in the α-B group than in the mirabegron group, as indicated by an OR of 1.51 (95% CI: 1.05-2.16; p = 0.03) in the meta-analysis. However, no significant differences were found between the α-B group and the mirabegron group for SET (MD: 1.20; 95% CI: -2.71 to 5.10; p = 0.55) [33].

For the secondary outcome, the mean expulsion interval and the amount of analgesia used were evaluated. For the SET, a pooled MD of 0.16 (-6.06 to 6.38) (p = 0.001), with I² = 97% (indicating very high heterogeneity), was obtained in the mirabegron subgroup, indicating that alpha-blockers significantly improve the SET. To the contrary, the tadalafil subgroup showed a pooled MD of -2.08 (-3.14 to -1.02) (p = 0.56), with heterogeneity of 0%. This means that the MD is significantly better for tadalafil over the alpha-blockers, and the results are statistically significant with high consistency between studies.

Sun et al. conducted a similar meta-analysis to evaluate the efficacy and safety of tadalafil compared to tamsulosin. A total of 17 studies were included, and data were measured through random- or fixed-effect models. The heterogeneity between studies was assessed by the I² test statistic. A shorter expulsion time was observed with a mean of -1.98 and 95% CI of 3.08-0.88 (p = 0.0004) that favored tadalafil significantly over tamsulosin [34].

Several limitations were encountered in our systematic review and meta-analysis. First, the heterogeneity of the results was high because two different drugs were evaluated, which could have affected the results as they have different mechanisms of action and bioavailabilities. Only English-based articles were screened, which could introduce language bias. Furthermore, in many studies, a small sample size was taken, with the duration of treatment being too short. Most of the RCTs were single-centered, which means there is a high risk of selection bias, and this limits the generalization of the results to a much broader population. This can be prevented by conducting larger multicenter prospective trials.

## Conclusions

The meta-analysis proved that tadalafil showed a trend toward improved outcomes compared to alpha-blockers in the MET of distal ureteric stones of <10 mm, but results are limited by the high heterogeneity and small number of studies. Tadalafil has a higher SER and a lower expulsion time and requires a reduced amount of analgesia. On the contrary, alpha-blockers are still better than mirabegron, but given that the certainty level of evidence is low to moderate, further research is required in the near future.
